# Comparative analysis of regulating characteristics between air-ring flow regulating valve and center butterfly valve

**DOI:** 10.1371/journal.pone.0251943

**Published:** 2021-05-19

**Authors:** Shixian Wu, Heqing Liu, Yongping Chen

**Affiliations:** 1 School of Resource, Environment and Safety Engineering, Hunan University of Science and Technology, Xiangtan, China; 2 Department of Energy Engineering and Building Environment, Guilin University of Aerospace Technology, Guilin, China; China University of Mining and Technology, CHINA

## Abstract

In this study, a novel air-ring flow regulating valve was proposed to reduce the flow resistance caused by valve structural pressure drop in fluid transportation pipeline system. The regulating characteristics at different valve openings were analyzed by numerical method and the results were compared with the center butterfly valve which is most widely applied in fluid transportation pipeline system. Besides, an experimental system was designed to validate the numerical model in the present study. The results indicated that the simulation results agree well with experimental data. The resistance coefficient of the air-ring flow regulating valve is smaller than that of the center butterfly valve when the valve opening is greater than 67%, and the resistance coefficient is reduced by up to 100% as the valve is fully opened. Both valves maintain approximately equal percentage flow characteristics, the deviation in relative flow coefficient is small. In addition, the wall shear stress of the air-ring flow regulating valve is much smaller than that of the center butterfly valve at the same valve opening, and the maximum velocity in the pipeline system is always smaller than that of the center butterfly valve, which significantly reduces valve surface abrasive erosion and thus prolongs its service life.

## 1. Introduction

The regulating valves are important components in the fluid transportation pipeline system. The flow field near the valve will become complicated when the fluid passes through the valve flow passage, especially when the valve is working at a small opening, which will cause both valve and pipe to be seriously damaged and cause a large number of energy loss in the fluid transportation pipeline system [1–4]. Therefore, it is of great practical significance to master the flow characteristics of the fluid in the throttling process of the regulating valve and optimize the design and operation of the pipeline system according to the characteristics. Based on this, the research on the regulation characteristics of regulating valves (butterfly valves, ball valves, globe valves, diaphragm valves, etc.) becomes an important research topic in the field of fluid transportation pipeline system [5–10].

It is well known that a butterfly valve is widely used in the industrial pipeline systems because of its smaller pressure loss and simple structure [11–13]. In the past decades, numerous researchers have focused on the optimization of butterfly valve structure and the analysis of flow characteristics. Kimura et al. [14] established the theoretical prediction equations for the pressure loss coefficients of the butterfly valve, and found that the theoretical predictions and experimental values were in good agreement. Kim et al. [15] conducted preliminary numerical simulation to analyze the internal flow field of the butterfly valve, but did not quantify the regulating characteristics. Corbera et al. [16, 17] used genetic algorithms to optimize the design of butterfly valve plates to improve the regulating characteristics of butterfly valves. Considering that the fluid contains particles, Liu et al. [18] found that particle erosion was easy to occur in the front of the butterfly valve plate while cavitation was easy to occur in the rear of the valve plate, which provides guidelines for reducing the butterfly valve plate erosion. Cui et al. [19] improved the butterfly plate structure to reduce the resistance coefficient, the results show that when the valve opening is 100%, the resistance coefficient decreases by 12%. Sun et al. [20] found that an increase in the roughness height significantly increased the frictional pressure drop. Liu et al. [21] changed tri-eccentric butterfly from a full-axis type to double-end-axis, and reduces the valve pressure drop at a small opening, but it doesn’t work when the valve is fully opened. Although the above studies have improved the regulating characteristics of the butterfly valve to a certain extent, however, the structural pressure drop cannot be completely eliminated for the condition of large valve opening, that is, the valve stem and the valve plate in the butterfly valve assembly are always in the flow field, which obstructs the fluid flow.

Considering the above problems of butterfly valves, in this paper, a new air-ring flow regulating valve was proposed. The structure of the valve is simple, and the flow passage is not blocked when the valve is fully opened, and thus its pressure drop can be ignored. In order to comprehensively evaluate the performance of air-ring flow regulating valve, the regulating characteristics at different valve openings were analyzed by numerical method and compared with the typical central butterfly valve. An experimental system was also built up to validate the numerical model. This work provides a theoretical guidance for the design and application of the air-ring flow regulating valve.

## 2. Valve model description

### 2.1 Geometry models of two regulating valves

**Fig 1:**
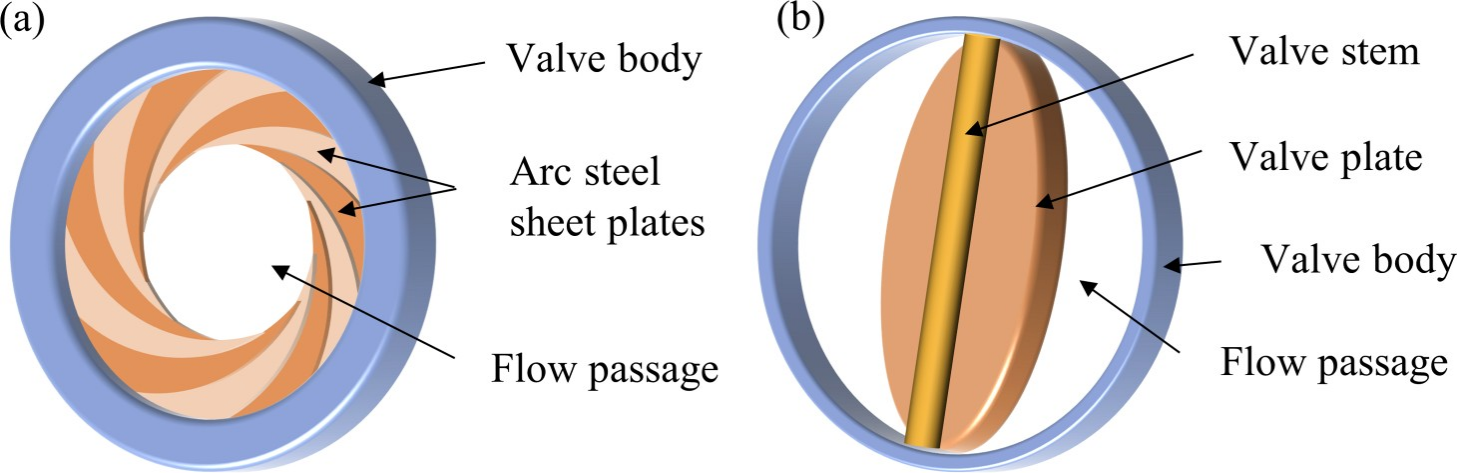
Three-dimensional simplified geometric models of two regulating valves. (a) Air-ring flow regulating valve. (b) Center butterfly valve.

The air-ring flow regulating valve achieves the regulating of flow rate through opening and closing the multiple overlapping arc steel sheet plates as shown in Fig 1(A). The center butterfly valve consists of a valve body, a valve plate and a valve stem (see Fig 1(B)). The flow rate is regulated by changing the size of the flow passage area by the valve plate, which is different from the air-ring flow regulating valve mentioned above.

### 2.2 Definition of valve opening

**Fig 2:**
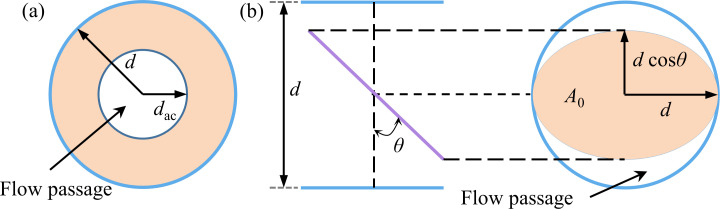
Valve structure parameters. (a) Air-ring flow regulating valve. (b) Center butterfly valve.

The air-ring flow regulating valve and the center butterfly valve are of two different structures. In order to unify the two valves openings, which is related to the flow passage area, the valves opening is defined as the area ratio of the flow passage area to the cross-section area of the pipe. Fig 2 shows the structural parameters of the two valves.

According to Fig 2, the two valves flow passage area is defined as follows:

$$A=\frac{\pi }{4}d^{2}$$ (1)

$$A_{\mathrm{ac}}=\frac{\pi }{4}d_{\mathrm{ac}}^{2}$$ (2)

$$A_{0}=\frac{\pi }{4}d^{2}cos\theta$$ (3)

$$A_{\mathrm{bc}}=A-A_{0}=\frac{\pi }{4}d^{2}(1-cos\theta )$$ (4)

The valve opening is defined as follows:

$$\eta_{\mathrm{ac}}=\frac{A_{\mathrm{ac}}}{A}={\left(\frac{d_{\mathrm{ac}}}{d}\right)}^{2}\times 100\%$$ (5)

$$\eta_{\mathrm{bc}}=\frac{A_{\mathrm{bc}}}{A}=(1-\cos\theta )\times 100\%$$ (6)

Where *d* is the diameter of the valve, m; *d*_ac_ is the flow passage diameter of the air-ring flow regulating valve, m; *θ* is the angle between the center butterfly valve and the radial direction, °; *A*, *A*_ac_, *A*_0_ and *A*_bc_ are the cross-sectional area of the pipe, the flow passage area of the air-ring flow regulating valve, the projection area of the center butterfly valve plate, and the flow passage area of the center butterfly valve, respectively, m^2^; *η*_ac_ and *η*_bc_ are the openings of the air-ring flow regulating valve and the center butterfly valve, respectively.

## 3. Numerical model

### 3.1 Physical model

**Fig 3:**
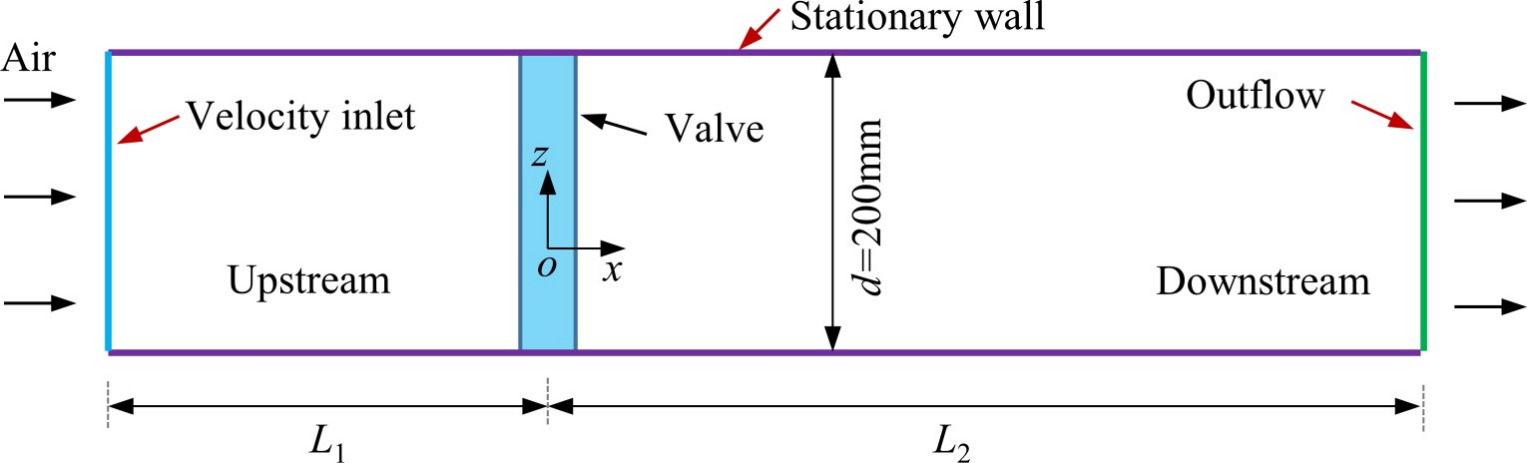
Schematic of the computational domain for two types of regulating valves.

The computational domain for two types of regulating valves is shown in Fig 3. To ensure the stability of the flow field, take the valve upstream pipe *L*_1_ = 6*d* (*d* is the diameter of pipe, *d* = 0.2m), the valve downstream pipe *L*_2_ = 12*d*, the computational domain length is 3.6m, and the valve is installed at *x =* 0m. It is worth noting that the diameter of pipe shown in Fig 3 corresponds to the practical application.

### 3.2 Mathematical model

The Standard *k*-*ε* model in CFD was widely employed to describe the flow characteristics in pipeline system and had been validated effectively [22–25]. Thus, the standard *k-ε* model was introduced in the simulation. The air flow in the pipe is governed by the momentum, continuity and turbulence equations. Their governing equations are expressed as follows:

1. Continuity conservation equation
  $$\frac{\partial \rho u_{i}}{\partial x_{i}}=0$$ (7)
2. Momentum conservation equation
  $$\frac{\partial (\rho u_{{}_{i}}u_{j})}{\partial x_{j}}=-\frac{\partial p}{\partial x_{i}}+\frac{\partial }{\partial x_{j}}\left[\mu \left(\frac{\partial u_{i}}{\partial x_{j}}+\frac{\partial u_{j}}{\partial x_{i}}\right)\right]-\frac{\partial (\rho u_{i}^{\prime }u_{j}^{\prime })}{\partial x_{j}}+F_{i}$$ (8)
3. *k* equation
  $$\frac{\partial (\rho ku_{i})}{\partial x_{i}}=\frac{\partial }{\partial x_{j}}\left[\left(\frac{\mu_{t}}{\sigma_{k}}+\mu \right)\frac{\partial k}{\partial x_{j}}\right]+\mu_{t}\left(\frac{\partial u_{i}}{\partial x_{j}}+\frac{\partial u_{j}}{\partial x_{i}}\right)\frac{\partial u_{i}}{\partial x_{j}}-\rho \varepsilon$$ (9)
4. *ε* equation
  $$\frac{\partial (\rho \varepsilon u_{i})}{x_{i}}=\frac{\partial }{\partial x_{j}}\left[\left(\frac{\mu_{t}}{\sigma_{\varepsilon }}+\mu \right)\frac{\partial \varepsilon }{\partial x_{j}}\right]+\frac{C_{1}\varepsilon \mu_{t}}{k}\left(\frac{\partial u_{i}}{\partial x_{j}}+\frac{\partial u_{j}}{\partial x_{i}}\right)\frac{\partial u_{i}}{\partial x_{j}}-C_{{}_{2}}\rho \frac{\varepsilon^{2}}{k}$$ (10)
  Where *ρ* is the air density, kg/m^3^; *u*_*i*_ and *u*_*j*_ are the air velocity components, respectively, m/s; $u_{i}^{\prime }$ and $u_{j}^{\prime }$ are the fluctuating velocity, respectively, m/s; *p* is the pressure on the air microelement, Pa; *F*_*i*_ is the body force per unit volume on the microelements, N/m^3^; *μ* is the dynamic viscosity, Pa·s; *μ*_*t*_ is the turbulent viscosity, Pa·s, where *μ*_t_ = *ρC*_*μ*_*k*^2^/*ε*; *k* is the turbulence kinetic energy, m^2^/s^2^; *ε* is the turbulence dissipation rate, m^2^/s^3^; *σ*_*k*_ and *σ*_*ε*_ are the turbulent Prandtl numbers for *k* and *ε*, respectively. According to the recommendations of Launder et al. and subsequent experimental verification [25, 26], the model coefficients are given by *C*_1_ = 1.44, *C*_2_ = 1.92, *C*_*μ*_ = 0.09, *σ*_*k*_ = 1.0, *σ*_*ε*_ = 1.3.

### 3.3 Calculation settings

The boundary conditions of different parts in the physical model can be founded in Fig 3. The air was flowed into the left side of the physical model under the velocity inlet boundary conditions, the inlet velocity *u* = 18m/s, and it is assumed to be uniformly distributed on the inlet surface. The pipe and valve in the model were set as stationary and no slip wall. The air outlet at the right of the model was set as outflow. In this work, the influence of gravity on the flow field was ignored. The SIMPLE algorithm was employed to decouple the pressure-velocity. The second order upwind scheme was used to solve the turbulence and momentum equations. The convergence accuracy and average air mass flowrate at the air outlet was monitored to estimate whether the convergence was achieved or not, the convergence accuracy is set as 10^−4^.

## 4. Experimental method

**Fig 4:**
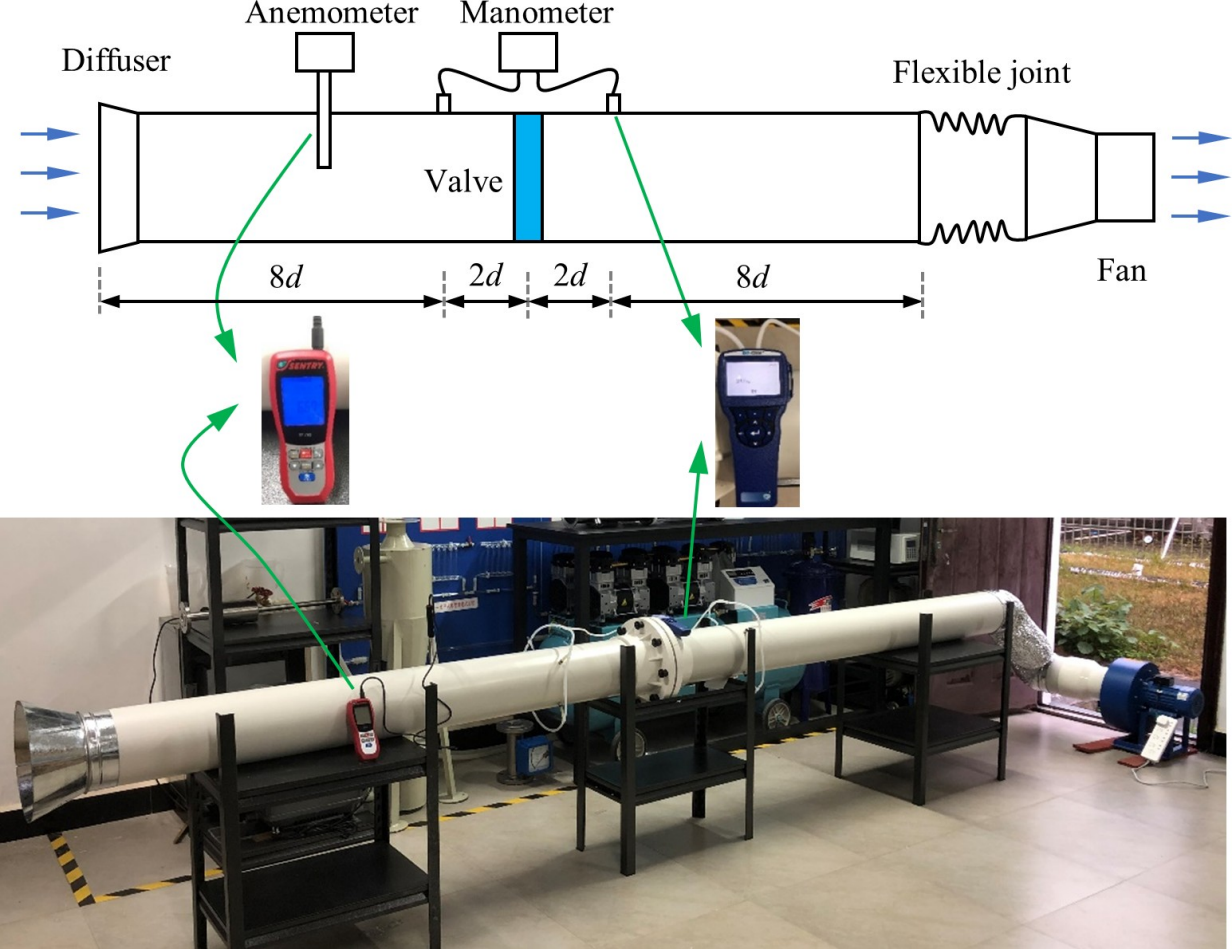
Schematic diagram of the experimental system.

An experimental system was designed and built up to validate the numerical model. The schematic diagram of the experimental system is shown in Fig 4. The main section of the system consists of a diffuser, wind pipe, air-ring flow regulating valve, flexible joint and fan. The airflow is induced by a fan at the end of the pipe for which the fan flow rate is controlled by a frequency inverter. Airflow velocity in the pipe is measured by using a thermal anemometer (sentry ST732), which is inserted upstream of the valve. The pressure drop of the valve is measured by using a manometer (TSI5825). To reduce the error of the experiment, four monitoring holes are set on the pressure monitoring section, which are connecting with the hose, and converge at one point to connect with the manometer. Meanwhile, the pressure drop was repeatedly measured five times, and the average values were calculated. In order to obtain a stable pressure value, the pressure sensors were installed at 2*d* upstream and 2*d* downstream of the valve, respectively. The diameter of the pipe is 0.2m.

## 5. Results and discussion

Numerical and experimental methods were used to analyze the regulating characteristics of the air-ring flow regulating valve and the center butterfly valve. The main values of parameters used in the numerical simulation and experiment are listed in Table 1.

**Table 1:** Values of parameters used in the numerical simulation and experiment.

Case	*η* / %	*d*_*ac*_ */ d*	*θ* / °
1	90.25	0.95	84.45
2	81	0.9	79.09
3	72.25	0.85	73.93
4	64	0.8	68.94
5	56.25	0.75	64.09
6	49	0.7	59.37
7	36	0.6	50.24
8	25	0.5	41.43
9	16	0.4	32.88
10	9	0.3	24.50

### 5.1 Model validation

#### 5.1.1 Grid independence

The numerical simulation solution of fluid flow problem mainly depends on the mesh quality [27]. To ensure that the results presented in this paper are independent of number of cells considered for the simulations, four different grid schemes were performed for both valves. The parameters of different grid schemes for the computational domain are tabulated in Table 2.

**Table 2:** Parameters of different grid schemes.

Air-ring flow regulating valve	Grid scheme	Case1	Case2	Case3	Case4
	Number of cells	360684	977588	1417350	1850958
Center butterfly valve	Grid scheme	Case1	Case2	Case3	Case4
	Number of cells	418193	1040761	146967	1621734

**Fig 5:**
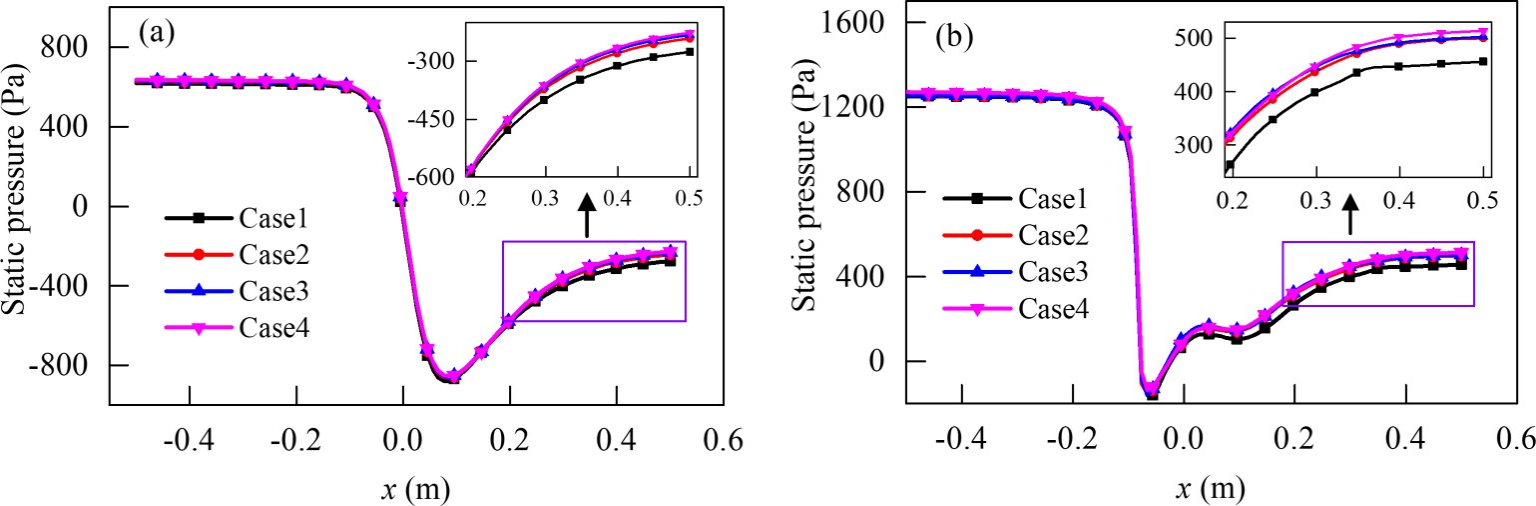
Static pressure along the *x* axis in the pipe at different grid schemes (*η =* 49%). (a) Air-ring flow regulating valve. (b) Center butterfly valve.

Fig 5 presents the static pressure along the *x* axis in the pipe at different grid schemes. Based on the values of finest grid scheme case 4, the deviations in the values of static pressure for the grid scheme case 2 and grid scheme case 3 are found to be small. However, for the grid scheme case 1, the deviation becomes large. The grid scheme case 2 is found to meet the requirements of both mesh-independence and computation time. Therefore, it was finally adopted for further simulation.

#### 5.1.2 Validation of numerical model

In order to examine the reliability of the present numerical model, we compare the predictions of the resistance coefficient of the air-ring flow regulating valve calculated by numerical model with the experimental data. The resistance coefficient can be defined as [28, 29]:

$$\xi =\frac{2\Delta P}{u^{2}\rho }$$ (11)

Where Δ*P* is the pressure drop of the valve, Pa; *u* is the fluid velocity in the pipe, m/s; *ρ* is the air density, kg/m^3^.

**Fig 6:**
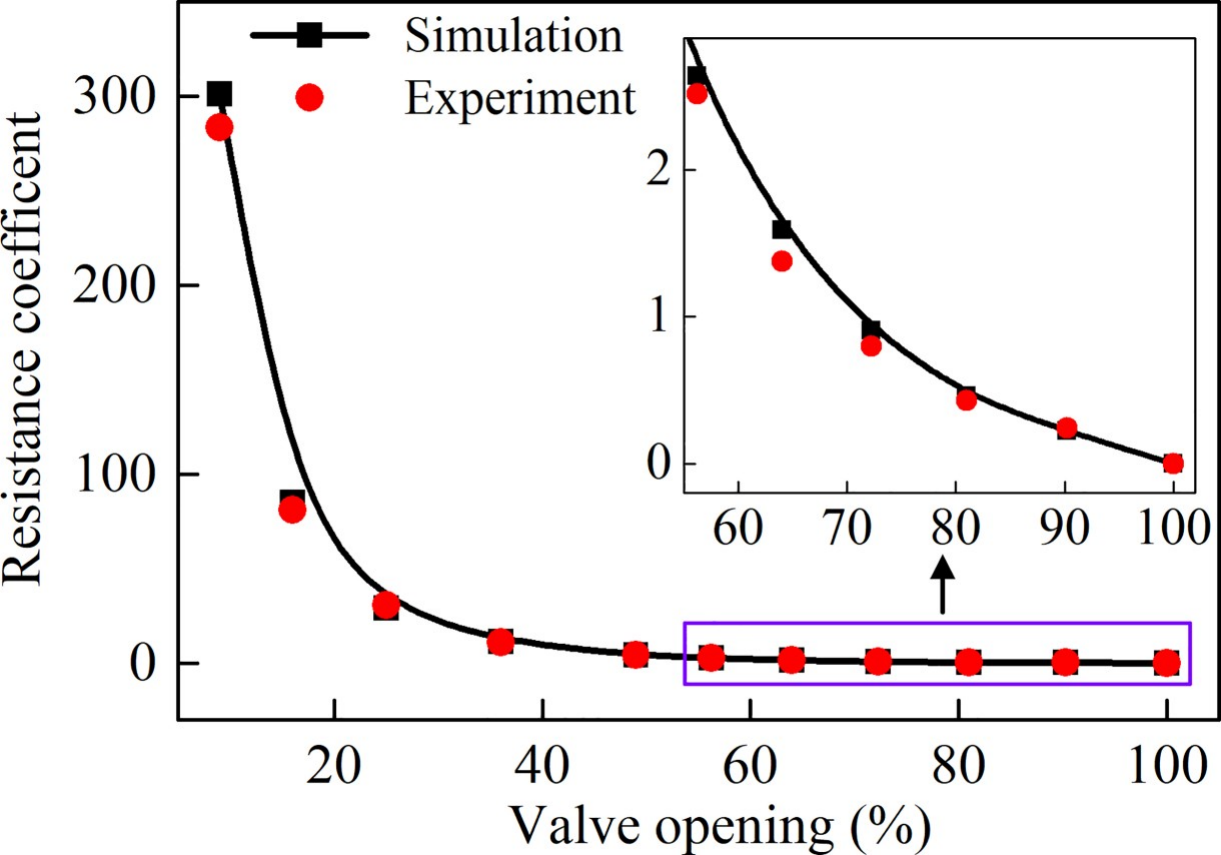
Comparison between the measured and simulated resistance coefficient of the air-ring flow regulating valve.

Fig 6 presents the simulation and experimental results of the resistance coefficient of the air-ring flow regulating valve. It is noted that the simulation results agree well with experimental data, which validates our simulation procedure. Therefore, the simulation results of the numerical model will be discussed in the following section.

### 5.2 Flow resistance characteristics analysis

#### 5.2.1 Resistance coefficient

**Fig 7:**
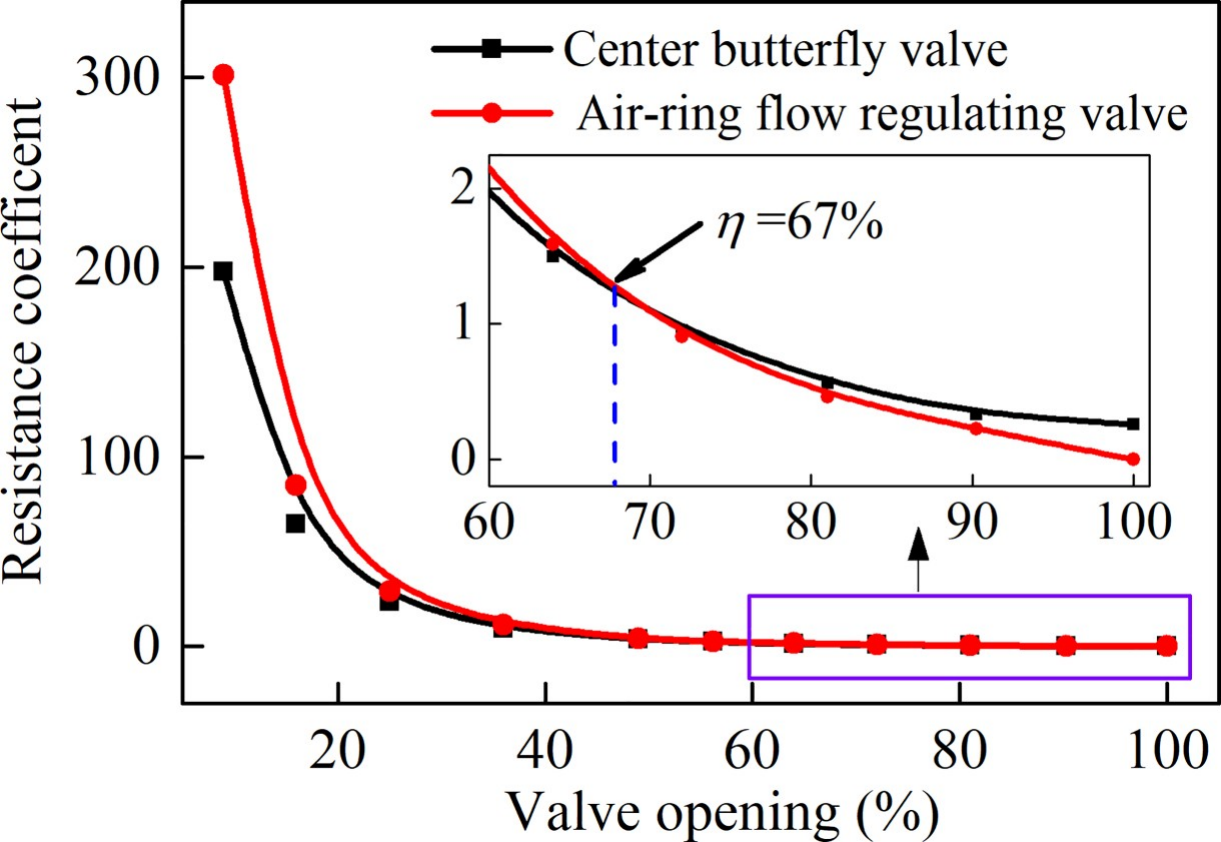
Comparison of resistance coefficient between the air-ring flow regulating valve and center butterfly valve.

Fig 7 presents the resistance coefficient of the air-ring flow regulating valve and center butterfly valve. It shows that the resistance coefficients of both valves increased exponentially with the decrement of valve opening. When both valves openings are decreased to 25%, the resistance coefficient values were increased dramatically, and the pressure drop of both valves are large. Therefore, regulating the valve opening too quickly will have a huge impact force on the valve. In addition, the resistance coefficient of the air-ring flow regulating valve is smaller than that of the center butterfly valve when the valve opening is greater than 67%, and the difference in resistance coefficient between the two valves reach maximum as the valve is fully opened. However, when the valve opening is less than 67%, the resistance coefficient of the air-ring flow regulating valve becomes larger than that of the center butterfly valve. With the further decrease of the valve opening, the resistance coefficient of the air-ring flow regulating valve will be gradually increased compared with the center butterfly valve.

Since the energy loss of fluid flow strongly depend on the valve resistance coefficient, a larger resistance means a greater energy loss. In order to quantitatively analyze decreased resistance coefficient percentage of the air-ring flow regulating valve over the center butterfly valve, the resistance coefficient descent factor (*φ*) is introduced and defined as:

$$\varphi =\frac{\xi_{1}-\xi_{2}}{\xi_{2}}\times 100\%$$ (12)

Where *ξ*_1_ is the resistance coefficient of the air-ring flow regulating valve, and *ξ*_2_ is the resistance coefficient of the center butterfly valve.

**Fig 8:**
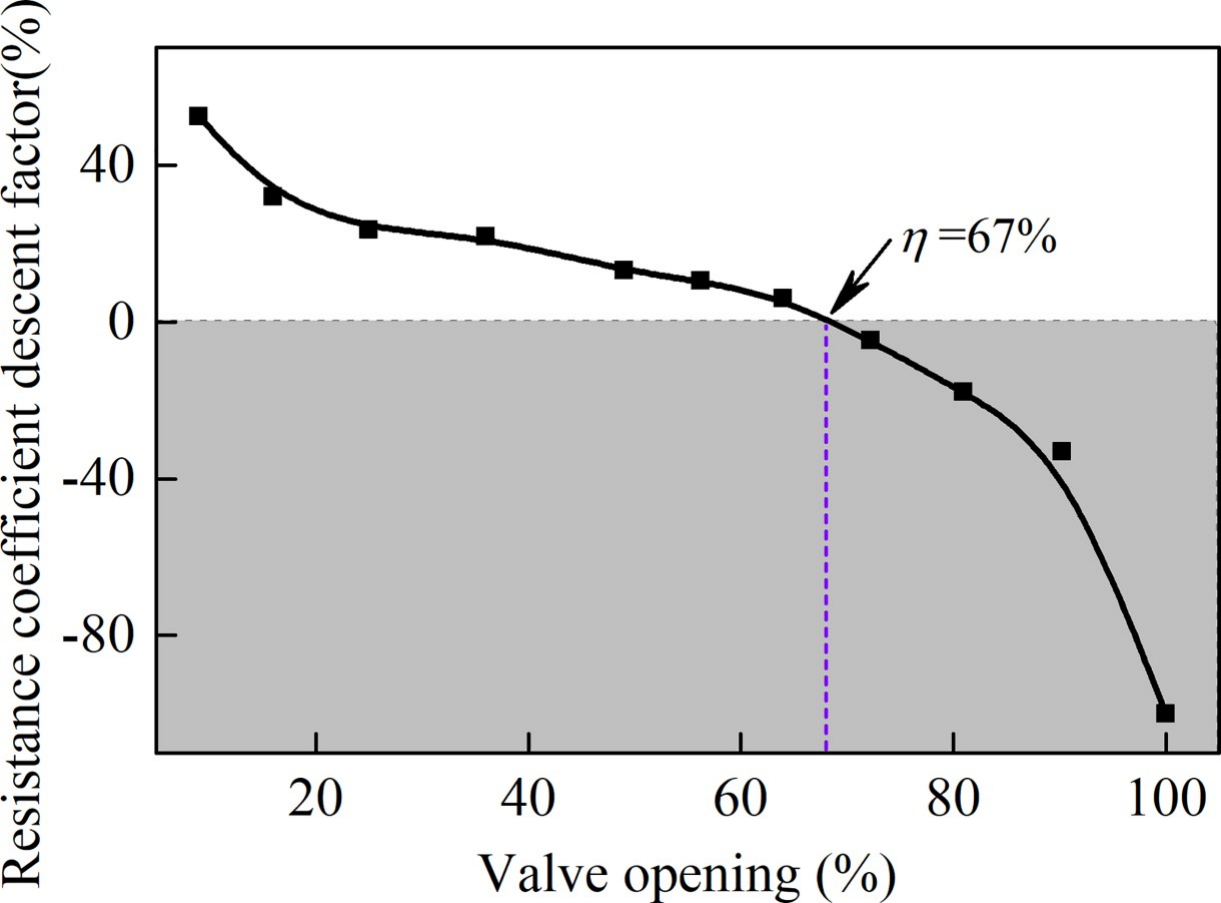
Resistance coefficient descent factor at different valve openings.

Fig 8 shows the resistance coefficient descent factor at different valve openings. From Fig 8, it can be seen that the resistance coefficient descent factor decreases with the increase of the valve opening. It is noted that when the valve opening is greater than 67%, the resistance coefficient ratio will be less than 0, which means a lower energy loss can be obtained with application of the air-ring flow regulating valve. When the valve opening increased from 67% to 100%, the resistance coefficient descent factor decreased from 0 to −100%. It is worth noting that the resistance coefficient is reduced by up to 100% as the valve is fully opened.

#### 5.2.2 Relative flow coefficient

The flow coefficient and relative flow coefficient can be defined as [28, 29]:

$$K_{v}=Q\sqrt{\frac{\rho }{\Delta P}}$$ (13)

$$K_{v}=\frac{K_{v}}{K_{v\max}}\times 100\%$$ (14)

Where Δ*P* is the pressure drop of the valve, Pa; *ρ* is the air density, kg/m^3^; *Q* is the fluid flow rate in the pipe, m^3^/h; *K*_*v*_, *K*_*v*max_ and *K* are the flow coefficient, maximum flow coefficient, and relative flow coefficient, respectively.

**Fig 9:**
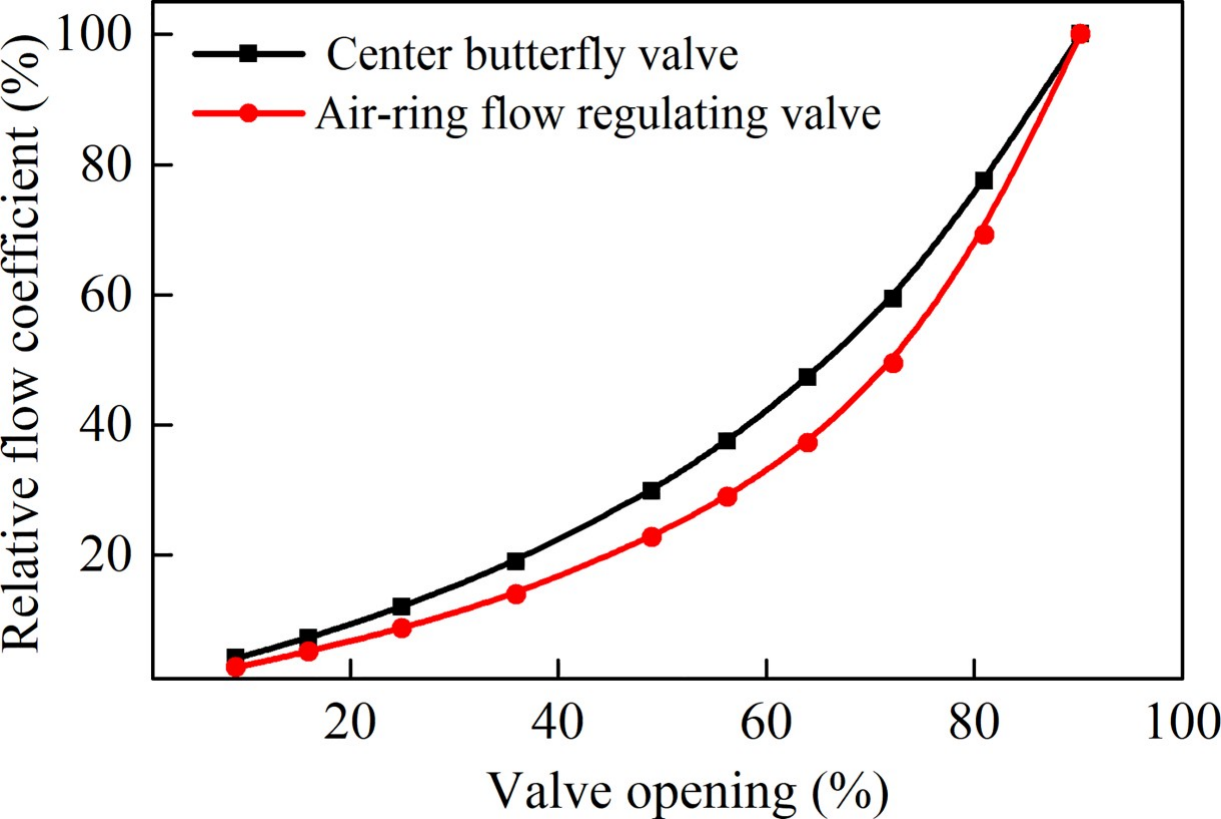
Relative flow coefficient of both valves at different valve openings.

For the convenience of analysis, based on the flow coefficient when the valve opening is 90.25%, a normalization process is performed to get the change curves of the relative flow coefficient with the valve opening, the results are shown in Fig 9. By comparison, the relative flow coefficient of the center butterfly valve is a little smaller than that of the air-ring flow regulating valve at the same opening. It is worth noting that both valves maintain approximately equal percentage flow characteristics, and the change of the flow rate is greater with large valve opening.

### 5.3 Wall shear stress analysis

**Fig 10:**
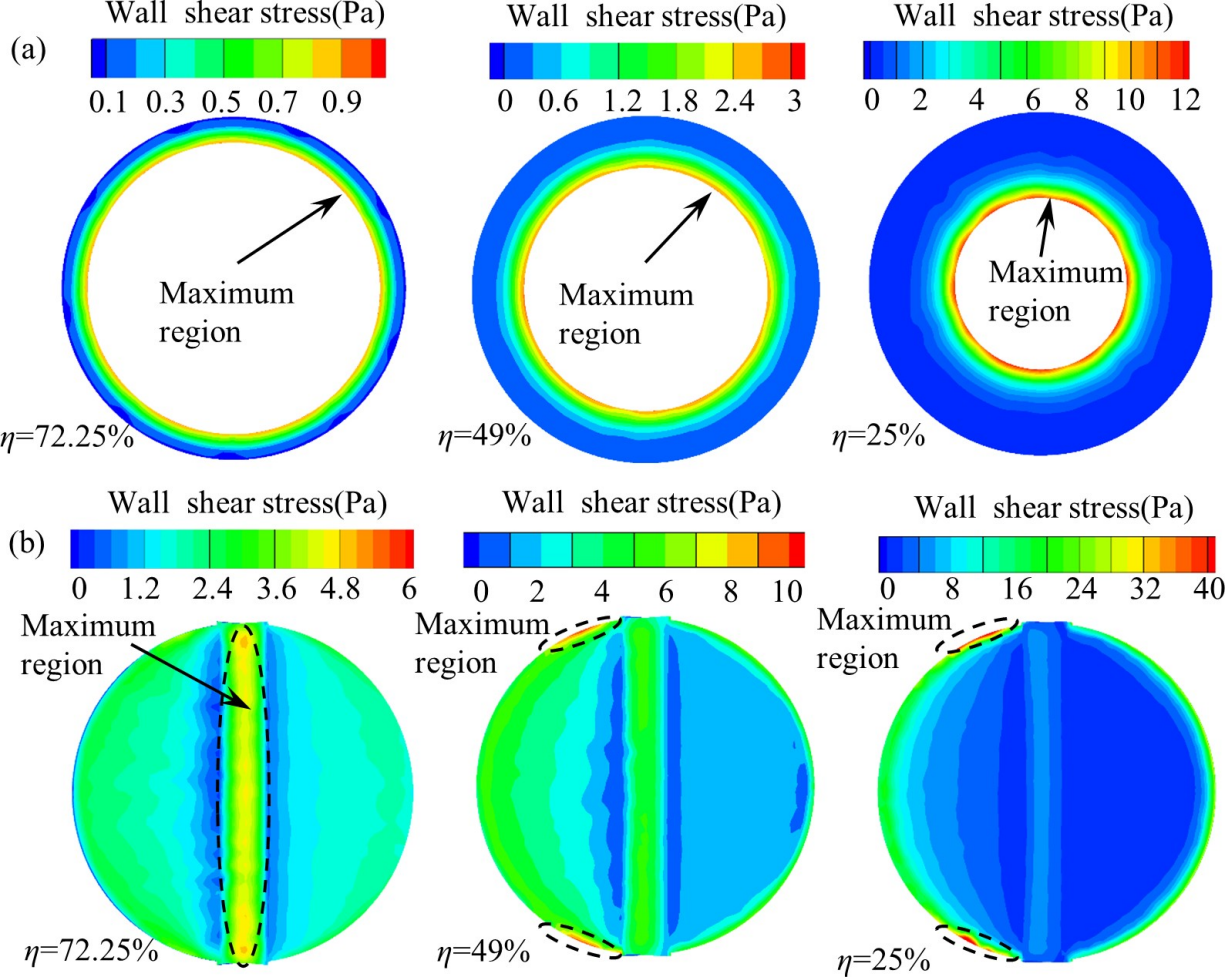
Wall shear stress distribution of the two valves at different valve openings. (a) Air-ring flow regulating valve. (b) Center butterfly valve.

Fig 10 shows the wall shear stress distribution of the two valves at different openings. It can be see that the wall shear stress of the both valves increases with the decrease of the valve opening. The wall shear stress of the air-ring flow regulating valve is mainly concentrated at the edge of the arc steel sheet near the flow passage, and the maximum region of wall shear stress is not affected by the valve opening (see Fig 10(A)) and Fig 10(B), the distribution region of the wall shear stress of the center butterfly valve has a large difference at different valve openings. When the valve opening is large (*η* ≥72.25%), the valve wall shear stress is mainly concentrated at the valve stem, whereas the valve wall shear stress is mainly concentrated at the valve plate and the valve stem when the valve opening is decreased to 49%, and reaches the maximum on both sides of the edge of the valve plate. When the valve opening further decreased (*η* ≤25%), the valve wall shear stress is mainly concentrated at the edge of the valve plate, and reached the maximum on both sides of the edge of the valve plate, while the wall shear stress on the valve stem is significantly reduced. Comparing Fig 10(A) and Fig 10(B), the wall shear stress of the air-ring flow regulating valve is much smaller than that of the center butterfly valve at the same valve opening.

**Fig 11:**
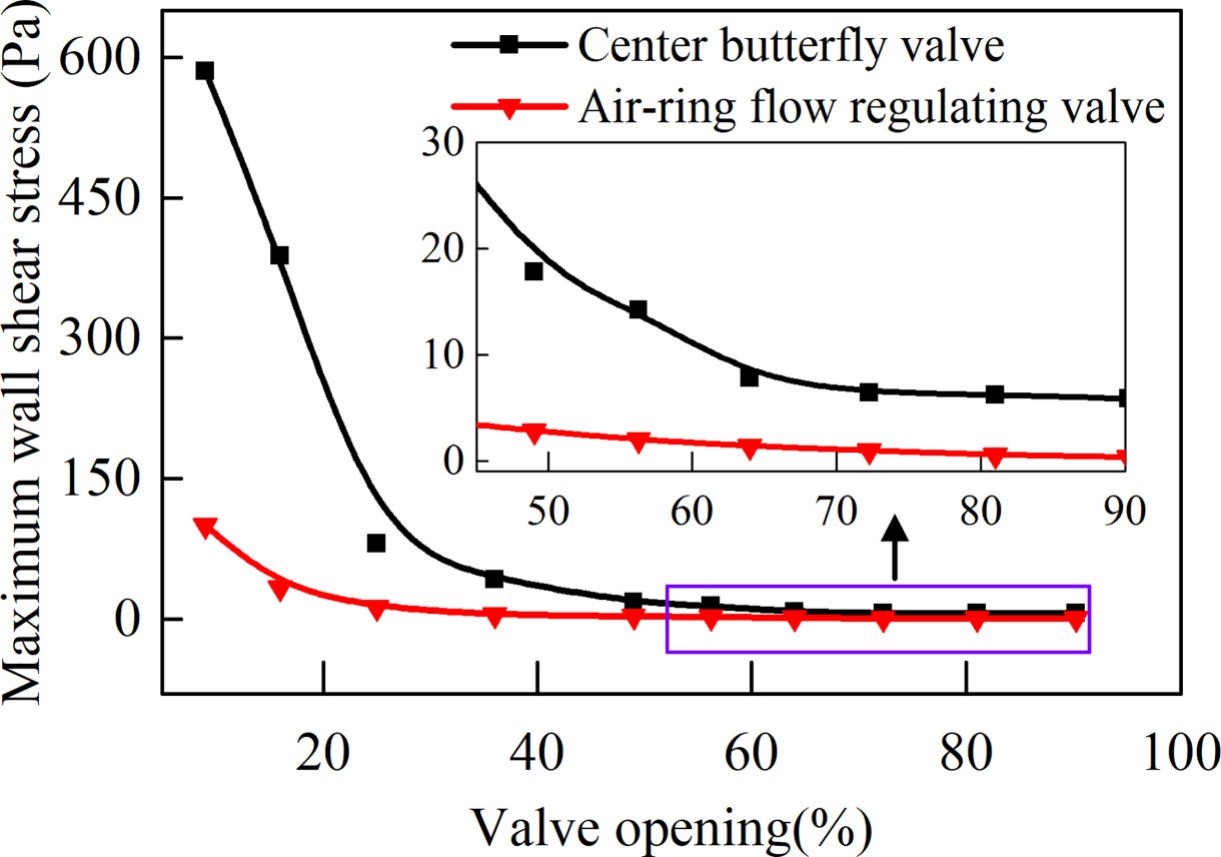
Maximum wall shear stress of two valves at different valve openings.

In order to quantitatively discuss the wall shear stress distribution rules of the two valves, the maximum wall shear stress *σ*_max_ at different valve openings is calculated and the results are shown in Fig 11. It can be seen that the maximum wall shear stress increases as the valve opening decreases, and the maximum wall shear stress of the air-ring flow regulating valve is always smaller than that of the center butterfly valve, and the difference between the two valves gradually increases with the decrease of the valve opening. Therefore, when the valve works at a small opening, the wall shear stress of air-ring flow regulating valve arc steel sheet will be significantly reduced compared to the center butterfly valve, thus prolongs its service life.

### 5.4 Flow field distribution

#### 5.4.1 Velocity field distribution

**Fig 12:**
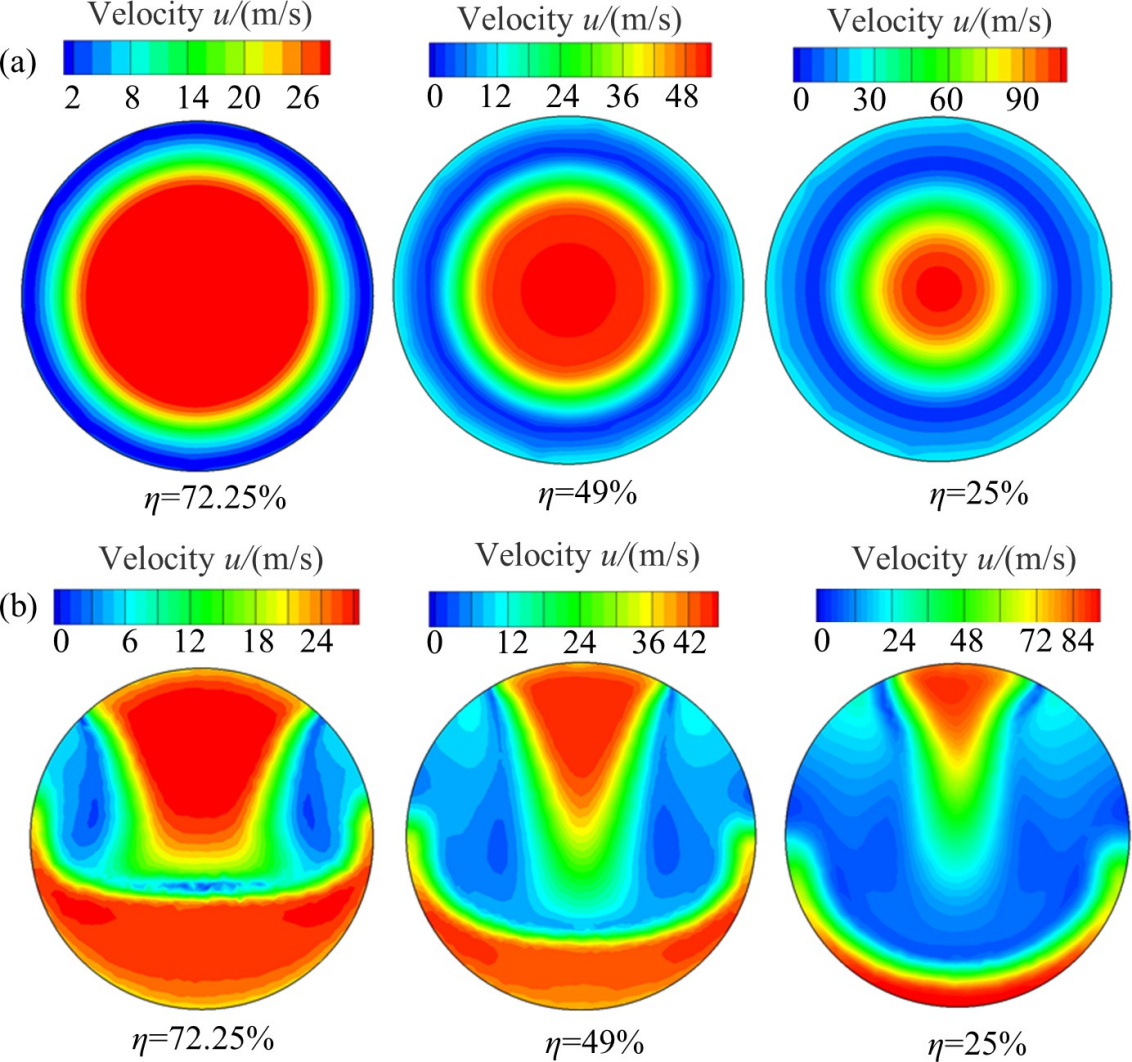
Velocity contours of *y*-*z* cross-section at different valve openings (*x* = 0.1 m). (a) Air-ring flow regulating valve. (b) Center butterfly valve.

Fig 12 shows the velocity contour of the *y*-*z* cross-section at different valve openings. For the two valves with different internal structures, there is a high velocity region and a low velocity region in the cross-section, but the velocity distribution of the two valves is significantly different. As shown in Fig 12(A), for the air-ring flow regulating valve, the high velocity region is in the center of the *y*-*z* cross-section, and a low velocity region appears around the pipe surface. As the valve opening decreases, the high velocity region gradually shrinks to the center of cross-section and decreases, the velocity increases significantly, and the low velocity region increases and gradually expands to the center of the cross-section. For the center butterfly valve (see Fig 12(B)), the high velocity region appears above and below the *y*-*z* cross-section and the low velocity region on both sides. As the valve opening decreases (*η* = 25%), the high velocity region gradually shrinks toward to the pipe surface, and the low velocity region gradually expands.

**Fig 13:**
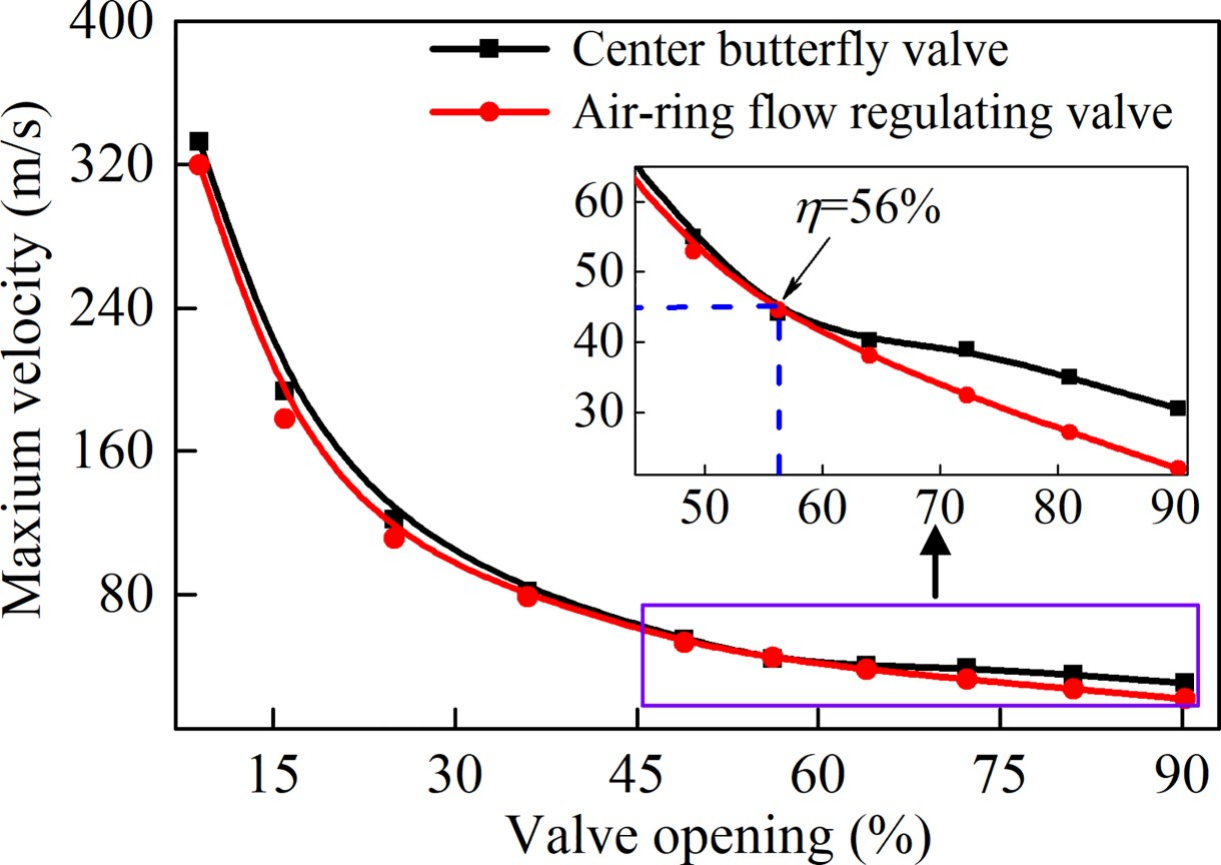
Maximum velocity of both valves at different valve openings in flow fields.

The maximum velocity *u*_max_ of both valves in flow fields at different valve openings are shown in Fig 13. It can be seen that the maximum velocity in flow fields increases as the opening decreases, and the velocity gradient increased dramatically as the opening decreases. Fig 13 also shows that the maximum velocity of the air-ring flow regulating valve is always smaller than that of the center butterfly valve, and the difference between the two (about 1%) is the smallest when the valve opening is approximately equal to 56%, which means that the air-ring flow regulating valve is always less forced by the fluid.

#### 5.4.2 Streamline distribution

**Fig 14:**
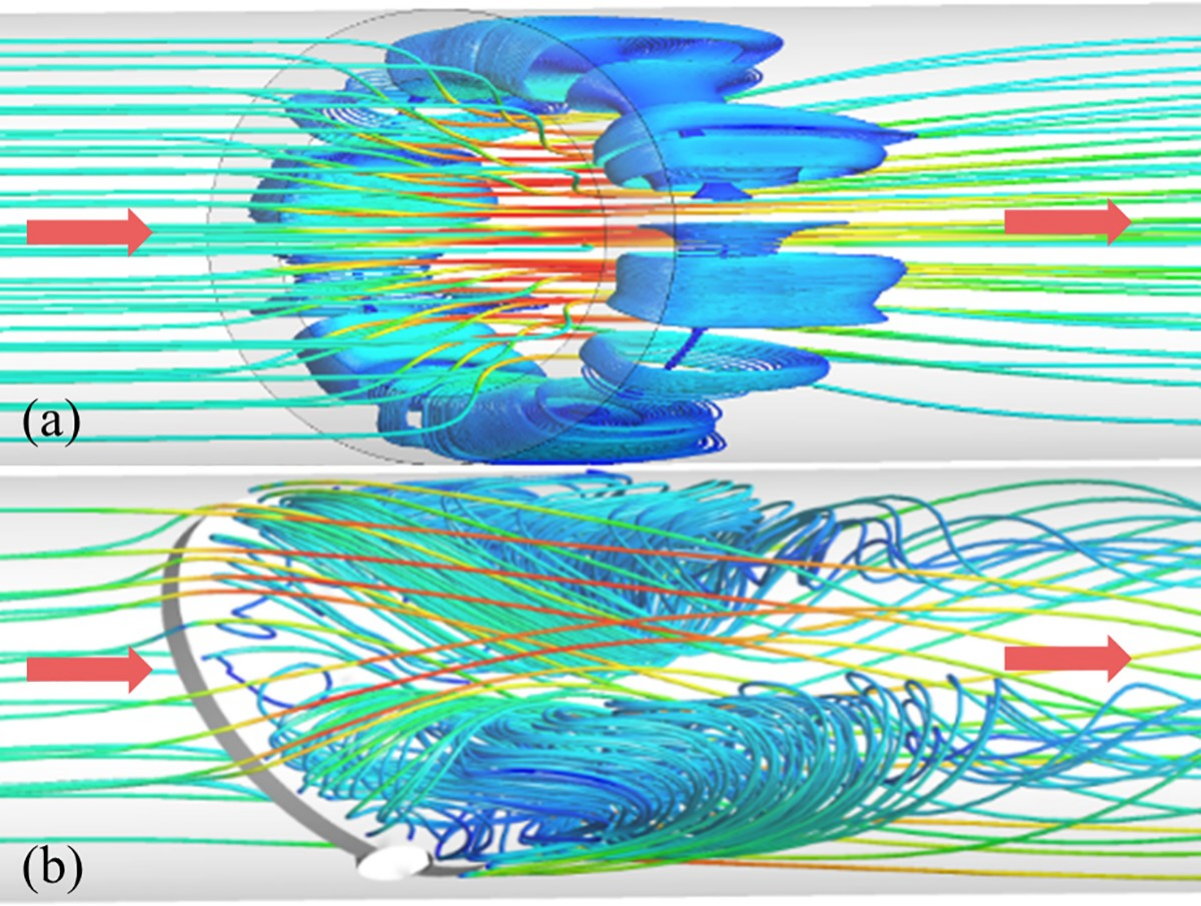
Streamlines distribution in the pipe. (a) Air-ring flow regulating valve. (b) Center butterfly valve.

Fig 14 presents the local streamlines distribution in the pipe of the two regulating valves. It can be seen that the vortex region of the air-ring flow regulating valve is mainly distributed around the surface of the downstream pipe, and the vortex region is distributed rotated symmetrically along the *x* axis (see Fig 14(A)) and Fig 14(B), the vortex region of the center butterfly valve is mainly concentrated on both sides of the downstream pipe, and the vortex region is distributed axially symmetrically along the *x* axis.

**Fig 15:**
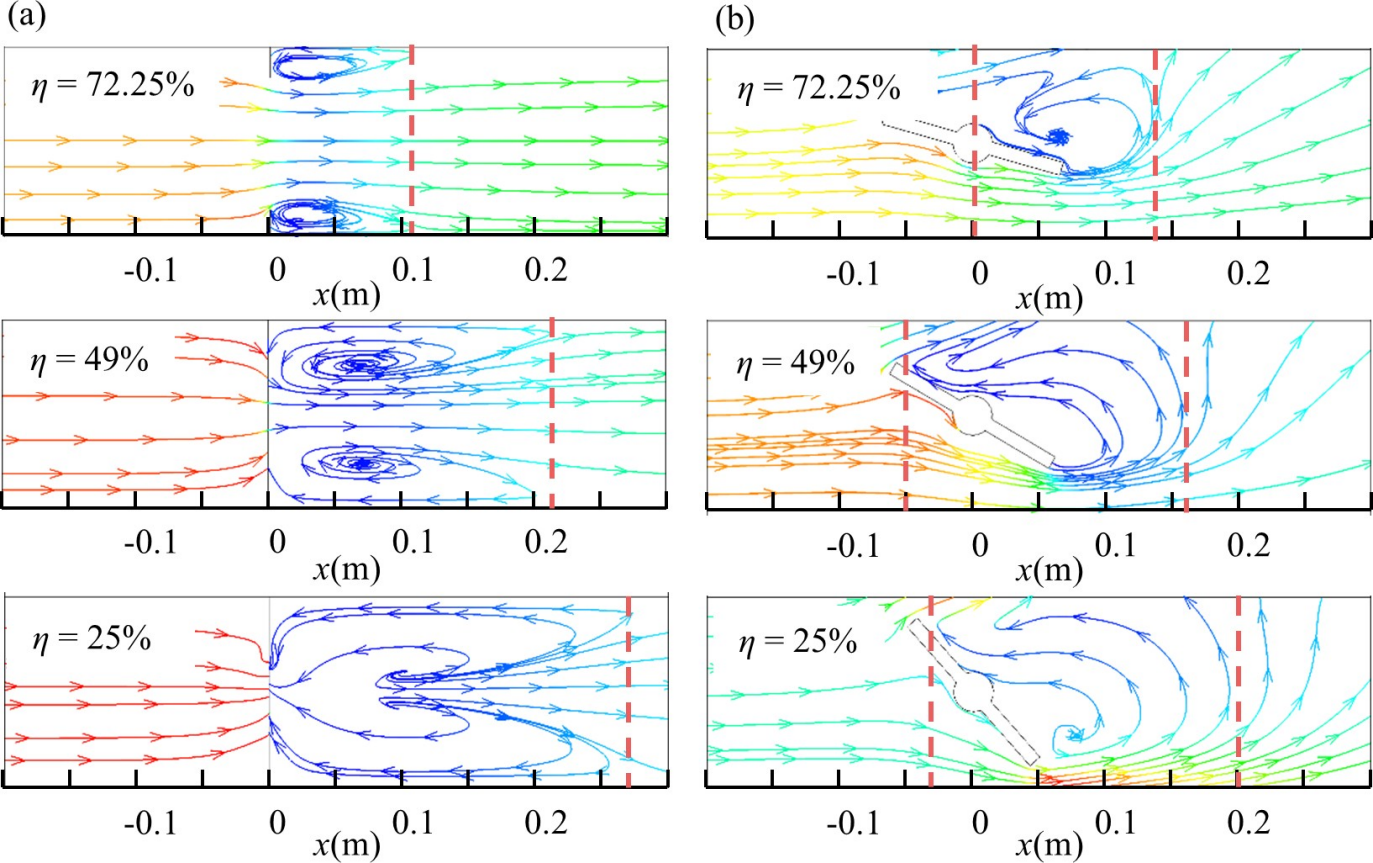
Streamlines distribution of *x*-*z* cross-section. (a) Air-ring flow regulating valve. (b) Center butterfly valve.

The generation of vortex will disturb the flow filed, and the fluid flow state becomes unstable, which will cause pressure drop for varying valve openings. In order to further investigate the influence of the valve opening on the vortex region, Fig 15 shows the streamlines distribution of the *x*-*z* cross-section of the two valves at different valve openings. The results show that the vortex region of the two valves gradually increases with the decrease of the valve opening. For the air-ring flow regulating valve (see Fig 15(A)), when the valve opening is large (*η* = 72.25%), the size of the vortex region is about 0.5*d*, and the vortex region of the center butterfly valve is about 0.7*d* (see Fig 15(B)). When the valve opening is small (*η* = 25%), the vortex region of the air-ring flow regulating valve is about 1.35*d*, and the vortex region of the center butterfly valve is about 1.15*d*. It can be seen that the influence range of the vortex of the air-ring flow regulating valve is smaller at a large valve opening, but at a small valve opening, the influence range of the vortex is larger. The larger the vortex region, the greater the pressure drop and energy loss. Therefore, the energy loss of the air-ring flow regulating valve will be smaller than that of center butterfly valve as the valve opening is large.

## 6. Conclusion

The present study proposed a novel air-ring flow regulating valve. Numerical and experimental methods were used to analyze its regulating characteristics and results were compared with the center butterfly valve. The simulations results were verified by the experimental data The results indicate that the resistance coefficient of the air-ring flow regulating valve is smaller compared with the center butterfly valve when the valve opening is greater than 67%, and the maximum energy-saving effect can be obtained when the valve is operated at fully open. Furthermore, both valves maintain approximately equal percentage flow characteristics, and the deviation in relative flow coefficient is small. The results also demonstrate that the wall shear stress of the air-ring flow regulating valve and the maximum velocity in the pipe are smaller than that of the center butterfly valve at the same valve opening. This characteristic will reduce the surface abrasive erosion of valve and the corresponding damage, and thus prolong the service life of the valve when using the air-ring flow regulating valve.

## Supporting information

S1 File Raw data. (XLSX)

S2 File (XLSX)
